# Mapping the Implementation Determinants of Second Dose Measles Vaccination in the World Health Organization African Region: A Rapid Review

**DOI:** 10.3390/vaccines12080896

**Published:** 2024-08-08

**Authors:** Abdu A. Adamu, Rabiu I. Jalo, Balcha G. Masresha, Duduzile Ndwandwe, Charles S. Wiysonge

**Affiliations:** 1 Polio Eradication Programme, World Health Organization Region Office for Africa, Djoue, Brazzaville P.O. Box 06, Congo; 2 Vaccine-Preventable Diseases Programme, World Health Organization Regional Office for Africa, Djoue, Brazzaville P.O. Box 06, Congo; masreshab@who.int (B.G.M.); sheyc@who.int (C.S.W.); 3 Department of Community Medicine, Faculty of Clinical Sciences, Bayero University Kano, Zaria Road, Kano P.M.B 3011, Kano State, Nigeria; rabiuibrahimjalo@yahoo.com; 4 Department of Community Medicine, Aminu Kano Teaching Hospital, Zaria Road, Kano P.M.B 3452, Kano State, Nigeria; 5 Cochrane South Africa, South African Medical Research Council, Francie van Zijl Drive, Parrow Valley, Cape Town 7500, South Africa; duduzile.ndwandwe@mrc.ac.za

**Keywords:** measles-containing vaccines, childhood vaccination, WHO African Region, implementation determinants, consolidated framework for implementation research, primary health care

## Abstract

The second dose of measles-containing vaccines (MCV2) has significant programmatic relevance in the current immunisation landscape because it serves as both an opportunity to reduce measles immunity gaps and strengthen second year of life vaccination platforms. However, MCV2 coverage remains suboptimal across countries in the World Health Organization (WHO) African Region and this puts a significant number of children at risk of morbidity and mortality from measles despite the availability of an effective vaccine. There is an urgent need to strengthen the implementation of MCV2 but this requires a thorough and systematic understanding of contextual factors that influence it. The literature that describes the determinants of implementation of MCV2 in a manner that adequately accounts for the complexity of the implementation context is scarce. Therefore, the purpose of this rapid review was to explore the implementation determinants of MCV2 in the WHO African Region using systems thinking. Literature search in two databases (PubMed and Google Scholar) were conducted. After screening, a total of 17 eligible articles were included in the study. Thematic analysis of extracted data was performed to identify the implementation determinants, after which they were mapped using the Consolidated Framework for Implementation Research (CFIR). A causal loop diagram (CLD) was used to illustrate the linkages between identified determinants. We found 44 implementation determinants across the five CFIR domains, i.e., innovation, outer setting, inner setting, individual, and implementation process. The majority of identified determinants are within the individual domain followed by the inner setting domain. The CLD showed that multiple contingent connections and feedback relationships exist between the identified implementation determinants within and across CFIR domains. The linkages between the implementation determinants revealed three balancing and reinforcing loops each. The findings suggest that implementation determinants of second-dose measles vaccination in the WHO African Region are complex, with multiple interconnections and interdependencies, and this insight should guide subsequent policies. There is an urgent need for further implementation research with embedded CLD in specific settings to inform the design of tailored systemic strategies to improve the implementation effectiveness of MCV2.

## 1. Introduction

Measles is a highly contagious paramyxovirus that is spread through breathing, sneezing, and coughing [1]. It has an estimated basic reproduction number of 12–18 [2], and most commonly affects children under the age of five years [3]. Measles infection is often characterised by high-grade fever, cough, redness of the eyes, runny nose, and rashes [4]. The infection can become complicated leading to croup, pneumonia, encephalitis, blindness, and death [4,5]. Before the advent of measles vaccination, nearly all children were infected and 2.6 million died each year worldwide [6,7]. Since the launch of the Expanded Programme on Immunization (EPI) by the World Health Organization (WHO) 50 years ago, measles cases and deaths have significantly declined [8]. In 2022, the estimated number of global measles-related cases was 9,232,288 (with 5,138,698 occurring in the WHO African Region) and deaths were 136,216 (with 85,417 occurring in the WHO African Region) [9]. Evidently, measles still remains a serious public health problem in the African region which disproportionately bears the majority of disease burden.

Measles vaccines are highly efficacious [10], and the vaccine effectiveness (VE) of MCV2 is estimated to be 94.1% (IQR: 88.3% to 98.3%) [11]. In spite of this, uptake has been persistently suboptimal across the WHO African Region [12]. The WHO recommends that children receive two doses of a measles-containing vaccine (MCV) [10]. For high burden settings, the first dose (MCV1) should be administered at 9 months of age while the second dose (MCV2) should be given at the age of 15–18 months [10]. In 2022, the WHO and UNICEF Estimates of National Immunization Coverage (WUENIC) data suggest that MCV1 and MCV2 coverage in the African region were 69% and 45%, respectively [12]. To achieve herd immunity for measles, coverage of at least 95% must be attained [10,13,14].

MCV2 has significant programmatic relevance in the current global immunisation landscape [15]. MCV2 has the advantage of reducing the population of children who are susceptible to measles among those who received the first dose but the vaccine did not generate sufficient protective immunity [10]. Protecting children from acquiring measles has a broader impact on the immunisation programme as emerging evidence suggests that measles infection can induce immune amnesia, making previously immunised individuals prone to diseases for which they have been vaccinated [16,17]. Also, efforts to improve MCV2 coverage serve as an opportunity to strengthen the second year of life (2YL) vaccination platform, as many countries are beginning to extend routine immunisation beyond infancy [18,19]. Moreover, the Immunization Agenda 2030 (IA2030) considers MCV2 coverage as one of the core indicators for measuring the performance and strength of immunisation programmes [15].

The large disruptive and cyclical measles outbreaks observed in multiple countries in the African region are indicative of persistent immunity gaps due to weak immunisation programmes [20,21,22], thus, strengthening the implementation of second-dose measles vaccination should be a programmatic imperative. An important first step towards improving MCV2 coverage is to understand the contextual factors that influence its implementation [23]. This is because contextual factors are responsible for the variation in the implementation effectiveness of health programmes including second-dose measles vaccination, determining their success or failure [23]. In the real world, contextual factors are constantly interacting with each other in a dynamic manner with emergent behaviours [24,25].

A recent review explored the predictors of MCV2 coverage in Africa and identified contextual factors such as awareness, educational status of caregivers, and distance to healthcare facilities among several others [26]. Building on this literature, it would be beneficial to use a systems thinking lens to foster a holistic understanding of the interconnectedness and interrelationship between the contextual factors that influence MCV2 implementation [27,28]. In this IA2030 era, it is essential to focus more on exploring the system’s behaviour of MCV2 implementation to enable sufficient consideration of feedback relationships in policymaking and innovation design [27]. A systems thinking approach can allow policymakers to focus on emergent behaviours rather than individual factors as it elucidates a “whole-of-system” view of facilitators and barriers that affect implementation [27].

In implementation science, contextual factors, whether facilitators or barriers are often referred to as determinants for ease of conceptualisation [28]. In addition, a “determinants framework” is the collective name for theoretical models that outline the structure underlying contextual factors [28]. These determinants frameworks are often categorised into domains and constructs to ensure a common understanding of the processes and mechanisms through which a group of factors influence implementation efforts [28]. One of the most commonly used determinant frameworks is the Consolidated Framework for Implementation Research (CFIR) [29]. This meta-framework has five domains and 48 constructs [30]. The domains include innovation, outer setting, inner setting, individual, and implementation process [30]. Using CFIR can contribute to a system-oriented exploration of the determinants of MCV2 implementation by highlighting their multilevel nature by domains [30]. However, CFIR does not show the interconnections and interdependencies that might exist between determinants within and across domains [28,30]. Interconnection means that determinants are linked with each other to form a whole, while interdependence means that determinants rely on and influence each other [27]. Both terms are commonly used in systems dynamics [27].

Systems thinking tools like the causal loop diagram can facilitate the illustration of the interconnections and interdependencies that exist between implementation determinants to unearth their collective behaviour [27]. Although the causal loop diagram emerged from systems dynamics, there has been a growing application in healthcare as stakeholders become more conscious of the behaviour of complex adaptive systems [31]. This qualitative systems mapping tool can expose feedback loops in the relationship between implementation determinants which can serve as leverage points for interventions [25,31].

To make progress towards measles elimination in the WHO African Region in line with the measles and rubella strategic framework 2021–2030 [32], and Immunization Agenda 2030 [15], countries need to attain and maintain the required threshold of second dose measles vaccination. This is particularly vital for reducing measles immunity gaps within countries and strengthening 2YL vaccination platforms to optimise access to vaccines provided beyond infancy, like the fourth dose of Diphtheria–Tetanus–Pertussis containing vaccine and malaria vaccines among others [18,33]. Efforts to strengthen the implementation of MCV2 in the WHO African Region require a thorough and systemic understanding of contextual factors that influence it. However, the literature that describes the determinants of implementation of MCV2 in a manner that adequately accounts for the complexity of the implementation context is scarce. Therefore, the objective of this study was to explore the implementation determinants of second-dose measles vaccination in the African region using a systems thinking approach.

## 2. Methodology

### 2.1. Study Design

A rapid review was conducted based on the guidance of the Cochrane Rapid Review Methods Group [34]. A rapid review simplifies evidence generation for stakeholders by excluding some methods of a traditional systematic review [34]. This knowledge synthesis methodology was used to produce a quick synthesis of available evidence on factors influencing second-dose measles vaccination in countries within the WHO African Region [35]. This methodology is advantageous because it can be conducted within a shorter period of time compared to a traditional systematic review [36]. A broad research question was used to ensure that many relevant publications were considered. The research question was: “What are the implementation determinants that influence second dose measles vaccination in the WHO African Region and how do they interact with each other?”

### 2.2. Search Strategy

On 3rd February 2024, a comprehensive online search of two databases, PubMed and Google Scholar, was performed to find published studies that reported on factors that affect second-dose measles vaccination in the WHO African Region. A detailed search strategy was developed. In the search strategy, keywords were combined with Boolean operators. In addition, truncations were used where necessary to broaden the search and improve the sensitivity of the search strategy. For PubMed, Medical Subject Headings (MeSH) were specified for some keywords so that the search can return all references that are indexed to them. Also, the “All Fields” option was used for some keywords so that the search could return all references where the term appeared. The search terms used are as follows: (MCV2 [All Fields] OR “second dose measles vaccin*” [All Fields] OR “second-dose measles vaccin*” “Measles-Mumps-Rubella Vaccine” [Mesh] OR “measles virus vaccin*”[tw] OR “Measles-Rubella Vaccine” OR “measles immunis*”[tw] OR “measles immuniz*” [tw] OR “measles vaccin*”[tw]) AND (uptake OR use OR utiliz* OR access* OR accept* OR refus* OR willing* OR hesitancy OR program* OR strateg* OR factor* OR implement* OR determinant* OR introduc* OR bottleneck OR constraint* OR facilitat* OR barrier OR enable* OR drive*). The search was geographically restricted to countries in the WHO African Region on PubMed. The search string was adapted for each database. However, no language or date restriction was applied.

### 2.3. Inclusion and Exclusion Criteria

To guide the formulation of the eligibility criteria for this study, the “Sample, Phenomenon of interest, Design, Evaluation and Research type” (SPIDER) framework was used. The criteria were as follows:

• Sample: Studies conducted in any country in the WHO African region;
• Phenomenon of interest: Studies that described the facilitators and barriers of second-dose measles vaccination;
• Design: Broad range of study designs including cross-sectional, longitudinal or experimental designs;
• Evaluation: Studies exploring the perspectives and experiences of different stakeholders involved in measles vaccination including caregivers, health workers, programme managers, cold chain officers, and community members among others;
• Research type: Mixed methods, qualitative and quantitative studies.

Studies were excluded if they were:

- Focused on other childhood vaccines;
- Conducted outside of the WHO African region.

### 2.4. Study Selection and Data Extraction

The outputs of the database search were combined, and duplicates were removed. About 40% of titles and abstracts of identified studies were screened by two authors for relevance. After this, one author proceeded to screen the remaining ones. The second author checked the studies that were excluded to ensure accuracy. The full texts of all relevant studies were obtained. One author screened them using the eligibility criteria and a second author checked the excluded studies for correctness.

Data extraction was performed using Microsoft Excel Office 365 to collect all the required information from included studies. This includes author name, year of publication, country of study, study population, study setting, study design and factors. This extraction was performed by one author and the second author checked the data form for completeness.

### 2.5. Data Analysis

The number of included studies was counted and a bibliographic analysis was performed to calculate the number of studies per year. This was presented using a radar chart. All the extracted factors were analysed using a qualitative thematic analysis [37]. This type of analytical framework can aid the identification of themes and patterns within data regarding second-dose measles vaccination [38]. The extracted factors were examined to gain a good sense of their themes and then organised according to how related they were. This led to the generation of descriptive themes which were further refined iteratively. Throughout the process, the linguistic reasoning of the original authors was maintained as much as possible to ensure that the meanings were not lost. All the factors were mapped to the domains and constructs of CFIR using deductive reasoning. The domains include innovation, inner setting, outer setting, individual, and implementation process [30]. For this study, innovation represents the measles vaccine that is being implemented. The inner setting is the place where measles vaccination are provided. The outer setting is where the inner setting exists, which is the health care system and community. Individuals include the innovation recipients and innovation deliverers. The implementation process refers to the strategies employed by the immunisation system to implement second-dose measles vaccination.

A complex system analysis was performed using the causal loop diagram (CLD) to qualitatively map the linkages and connections between the implementation determinants that influence second-dose measles vaccination. One author performed the initial mapping, which was then validated by the other authors. When constructing the CLD, the implementation determinants were the variables. Linkages were informed by descriptions provided in the original manuscripts and the experiences of the authors. Arrows were used to show the direction and influence between determinants, and their polarity was denoted using (+) and (−) signs. If change in a variable causes another variable to change in the same direction, then the polarity was said to be (+). But if change in a variable causes another variable to change in a different direction, then, the polarity was said to be negative (−). The feedback loops between variables could either be balancing (B) or reinforcing (R). A balancing (B) loop means that the direction of change between variables was countering each other. A reinforcing (R) loop means that the direction of change between variables is compounding, which can be vicious (negative consequences) or virtuous (positive consequences). The CLD was constructed using Vensim Personal Learning Edition (PLE) version 9.4.0 [39].

## 3. Results

**Figure 1:**
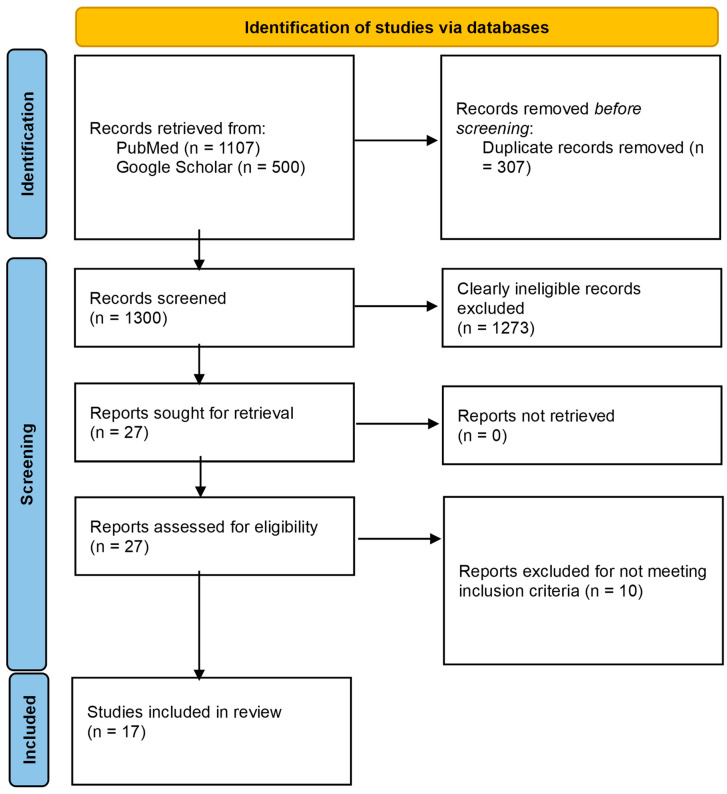
PRISMA flow diagram for the study.

The database search of PubMed and Google Scholar yielded 1107 and 20,800 records, respectively. For Google Scholar, only the first 500 records that were returned by the database (in order of relevance) were considered [40]. Following screening and eligibility assessment, 17 studies were included in this review. The study flow chart is presented in Figure 1.

### 3.1. Characteristics of Included Studies

**Figure 2:**
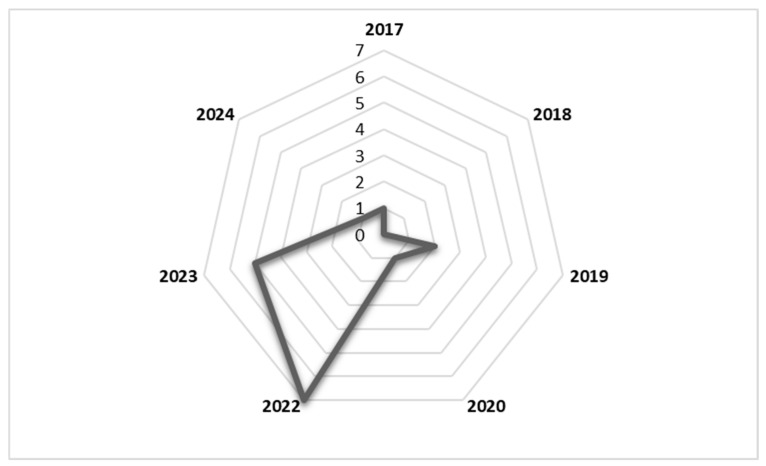
Radar chart showing number of publications per year from 2017 to 2024.

The literature included in this study was published between 2017 and 2024. As shown in the radar chart in Figure 2, the number of publications reporting factors affecting second-dose measles vaccination spiked in 2022 and 2023. The study design that was most commonly used in included studies was the cross-sectional quantitative design. The study population included caregivers, health workers, and immunisation programme managers, among others. Details of the included studies are shown in Table 1.

### 3.2. Implementation Determinants of Second Dose Measles Vaccination in the WHO African Region

A total of 44 implementation determinants that influence second-dose measles vaccination were identified and these determinants cut across all five CFIR domains as shown in Table 2. The number of determinants in each domain is as follows: innovation domain—1 (2.3%), outer setting domain—5 (11.4%), inner setting domain—11 (25%), individual domain—23 (52.3%), and implementation process domain—4 (9.1%). These determinants are multilevel, arising from the vaccine itself, individuals (such as children, caregivers, and health workers), the health system, and society.

As shown in Table 3, the implementation determinants of second-dose measles vaccination align with multiple CFIR constructs.

**Innovation domain:** This domain represents the measles vaccine itself. Only one determinant was identified which fell within the innovation cost construct.

**Inner setting domain:** This group of determinants influences the setting in which the second dose of measles vaccination is being implemented. They are related to constructs such as compatibility, access to knowledge and information, available resources and structural characteristics.

**Outer setting domain:** This group of determinants is at play in the external environment that surrounds the setting in which the second dose of measles vaccination is being implemented. They include local conditions such as socioeconomic status of the environment, political commitment and support and local attitudes originating from religion and traditional beliefs.

**Individual domain:** This group of determinants is related to individuals; the innovation recipients and innovation deliverers. Innovation recipients are those who directly or indirectly receive second-dose measles-containing vaccines. Factors within this construct are child and caregiver-related factors. Innovation deliverers are those that directly or indirectly deliver second-dose measles-containing vaccines. The factors within this construct are health worker-related.

**Implementation process domain:** This group of determinants is concerned with the strategies that are used to implement second-dose measles-containing vaccination. They cover constructs such as teaming, engaging and reflecting, and evaluating.

### 3.3. Dynamics of the Implementation Determinants of Second-Dose Measles Vaccination in the WHO African Region

**Figure 3:**
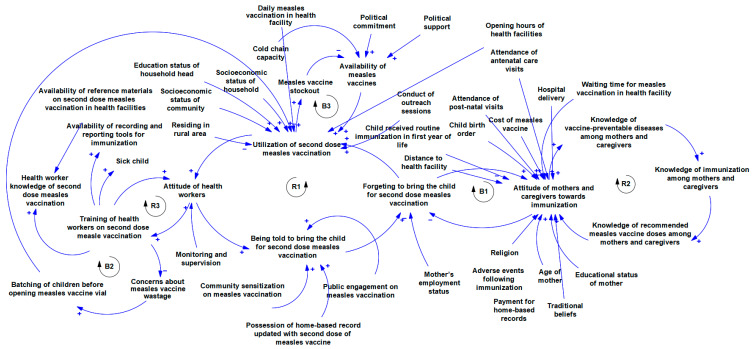
Causal loop diagram of the implementation determinants of second dose measles vaccination.

Figure 3 shows multiple contingent connections and feedback relationships between the implementation determinants of second-dose measles vaccination. There is a relationship between training health workers on second-dose measles vaccination and their attitude towards vaccination. Also, training is linked with the level of concern that they place on vaccine wastages and practices like batching of children before providing the measles vaccination which affects utilisation. There is a linkage between the attitude of health workers and the extent to which they remind mothers/caregivers to bring their children to the health care facility for a second dose of measles vaccination as this affects utilisation if mothers and caregivers forget to bring their children for second dose measles vaccination. The level of knowledge of mothers and caregivers about vaccine-preventable diseases is linked with their knowledge of immunisation in general and recommended doses of measles vaccines in particular all of which is connected with their attitude towards immunisation. The attitude of mothers and caregivers interconnect with how they forget to bring children for immunisation. Multiple determinants are linked to the attitude of mothers and caregivers towards immunisation and they include experience with immunisation services like waiting time, and experience with other essential health care services like antenatal care, postnatal care, and hospital delivery among others. Other connections are shown in the Figure 3.

## 4. Discussion

This rapid review aimed to explore the implementation determinants of second-dose measles vaccination in the African region using a systems thinking approach. A total of 44 implementation determinants of second-dose measles vaccination were identified across all five domains of CFIR, the majority of which are in the individual domain. These multilevel determinants of MCV2 implementation are related to the measles vaccine itself, individuals (i.e., caregivers, health workers, and other actors), health system (i.e., governance, information systems, workforce, service delivery and financing) and society. The implementation determinants were found to interact in a dynamic manner with several interconnections and interdependencies within and across domains, and feedback loops that are reinforcing and balancing. The findings confirm the complexity of the implementation determinants of second-dose measles vaccination in the WHO African Region.

This study innovatively used CFIR to guide the analysis of the implementation determinants of second-dose measles vaccination based on previous studies [30]. The advantage of using a theoretical framework to explore determinants is that it allows comparability across different settings [28]. The elucidation of the multilevel nature of these implementation determinants underscores the value of using an implementation science lens to guide context assessment.

The influence of implementation determinants on the implementation success or failure of evidence-based interventions in healthcare is well documented in implementation science literature [28,30,57]. This notion applies to second-dose measles vaccination as well, as such, policymakers need to understand that measles vaccine availability within a system does not necessarily guarantee uptake across settings. This is why insights on the implementation determinants of second-dose measles vaccination are crucial so that policymakers understand the causes of variation in implementation success, and use this knowledge to guide decision-making and action for optimising sustained uptake across diverse settings [58].

In this study, many of the implementation determinants that were identified are clustered in the individual and inner setting domains, and this highlights the critical importance of the behaviour of multiple actors and the health facility that is responsible for delivering the MCV2 in the African region. Efforts to strengthen the implementation of MCV2 can prioritise these domains, although systematic tailoring of strategies to specific contexts is needed to maximise demand and uptake. An advantage of CFIR is that its domains and constructs are linked to the Expert Recommendations for Implementing Change compilation which can ease the selection of evidence-based implementation strategies [59,60]. There are several examples of settings where stakeholders have used CFIR in this manner to improve and strengthen healthcare service delivery [60,61].

To further advance system-oriented approaches in healthcare, there has been a push for a paradigm shift towards systems thinking [62]. This is because implementation determinants interact with each other in a non-linear manner in the real world, and this necessitates non-reductionist analytic methods [23,28,62]. This epistemological belief guided this study, and to illustrate the complexity of the implementation determinants of second-dose measles vaccination, a CLD was used [25]. The CLD demonstrated that linkages exist between implementation determinants within and across the CFIR domains. This “whole-of-system” view of the implementation determinants of second-dose measles vaccination provides better clarity on the interconnections and interactions that produce emergent behaviours. Adopting this complexity lens propagated a more nuanced understanding of how determinants influence each other, especially the feedback loops that exist between them.

As shown in Figure 3, Loop R1 (health workers’ attitude loop) demonstrates a clear linkage between the attitude of innovation deliverers and the response of innovation recipients (caregivers). If utilisation is typically low, health workers are less likely to pay attention to measles vaccination. This decreases the rate at which caregivers are reminded to bring their children for a second dose of measles vaccination, and if mothers are not reminded, they are likely to forget, which in turn decreases utilisation. On the other hand, loop B1, which is the caregiver attitude loop, is balancing. A good attitude towards immunisation reduces forgetfulness to take the child for a second dose of measles vaccination, and when these caregivers use measles vaccination services, their attitude towards immunisation further improves. However, other determinants such as the birth order of the child, previous experience with the health system, distance to the health facility, and cost of vaccines also influence caregiver attitude. The knowledge loop (R2) shows that as the level of knowledge about vaccine-preventable diseases increases, knowledge about immunisation will also increase. Mothers and caregivers with good knowledge of immunisation will know the recommended measles vaccination for their child. And when mothers have good knowledge of the vaccine, their attitude improves. In turn, mothers and caregivers with positive attitudes towards immunisation will be more receptive to educational materials on vaccines and diseases, leading to better knowledge of vaccine-preventable diseases. Loops R2, B2, and R1 illustrate a knowledge–attitude–behaviour continuum with respect to second-dose measles vaccination. This continuum is well established in health promotion literature and serves as an important foundation for designing behaviour change interventions [63].

Loop R3 shows that proper training of health workers on measles vaccination reduces concerns about vaccination wastage, and decreased concern about wastage reduces the culture of batching children before opening measles vaccine vials, which in turn improves utilisation. Health worker training is also linked with attitude as illustrated in loop R3. As more health workers are trained on second dose measles vaccination, they will become more skilled at it, and this improves their attitude. As attitude towards second-dose measles vaccination improves, participation in training will increase. There is a delicate balance between measles vaccine availability and utilisation as shown in loop B3. As utilisation increases, available stock will be consumed leading to stockout, which in turn decreases availability. If vaccines are unavailable in the facility, utilisation will drop. As expected, political factors influence measles vaccine availability in the health facility. If there is political interest in measles vaccination, facilities will secure adequate stock. It is important to note that utilisation is affected by daily measles vaccination as well as facility opening time. If facilities provide daily measles vaccination and opening time is convenient for people in the communities, utilisation increases. To ensure that the vaccine is readily available in the health facility, factors such as cold chain capacity need to be strongly considered as well.

The multiplicity of the feedback loops in the dynamics of implementation determinants of MCV2 signals the need for the use of systemic innovations that target feedback loops to optimise performance [25]. For example, a commonly reported problem with measles vaccine delivery is that the vaccine vial has to be reconstituted, and if not used within 6 h, then, it will have to be discarded [64]. During this period, the vaccine cold chain needs to be maintained [64]. This study found that in some settings, to avoid wastage, health workers often batch children—usually 10—before opening a vial, and this affects utilisation [42,47]. This was illustrated in Loop B2. Given the widespread nature of this problem, it might be valuable to encourage stronger programmatic consideration for the use of smaller measles vaccine vial sizes among countries in the region while bearing in mind the logistical challenges that this can pose to the system [65]. There are countries in the WHO African region that have already tested the use of smaller measles vaccine vial doses and this is an opportunity for cross-country learning [66]. Furthermore, there is a need to improve consistency and adherence to national measles vaccine vial-opening policies in routine immunisation settings [67]. In addition, emerging innovations like microarray patches (MAP) for measles-containing vaccines can reduce this bottleneck in some settings [68]. MAPs are biomedical devices with micro projections that are capable of delivering the required vaccine dose into the dermis of the skin [68,69]. This novel technology can lessen measles-containing vaccine delivery barriers related to cold chain issues, as MAPs are designed to be more thermostable [64]. Moreover, since these patches are designed for single-dose use, they eliminate concerns about vaccine wastage [69]. In addition, MAP can be administered by people who are not healthcare workers.

Mothers’ and caregivers’ attitudes towards immunisation for measles and other vaccine-preventable diseases were found to influence the implementation effectiveness of second-dose measles vaccination as well. In particular, the mother’s age and childbirth order seem to be a recurring theme across multiple contexts, as several studies reported that utilisation was lower among young mothers and children of the first birth order [18,48,51,52,56]. This finding is important for policy as it necessitates the differentiation of behaviour change interventions for young mothers and older mothers. For example, an antenatal visit health education plan for primipara can emphasise second dose measles vaccination compared to that of multipara mothers. In addition, the primary health care system should enhance community engagement through the co-development of culturally acceptable messages that specifically target young primipara mothers with information about immunisation in the second year of life while also using the same platform to sensitise the same audience about antenatal care, hospital delivery, and postnatal care.

Interconnected inner setting implementation determinants such as waiting time for measles vaccination services, provision of measles vaccination services on a daily basis, and facility opening hours were commonly reported across different settings [42,47,51,53]. Considering the dynamics of these determinants vis-à-vis the broader system, there is a need to consider immunisation service pathway redesign to improve the experience of mothers who visit health facilities as part of the package of strategies for performance enhancement. The pathway redesign can focus on integrating immunisation into other healthcare services in the facility so that routine immunisation including measles vaccines can be administered to children at any service delivery point. So, rather than concentrate the flow of children to one (immunisation) point, routine immunisation delivery is re-engineered to decentralise service delivery across other points in the health care facility to improve efficiency and throughput (i.e., the number of children that are vaccinated in the healthcare facility), as well as caregiver satisfaction. However, when embarking on such pathway redesign, it is useful to embed quality improvement models like plan-do-study-act cycles, lean or agile [70,71,72].

Mothers and caregivers who missed the second dose of measles vaccination often reported that they were unaware of the need to return or forgot [41,42,47,51,53,56]. This indicated that defaulter tracking is also a crucial strategy that should be considered. Information technology can enhance this by aggregating data on the number of vaccine doses administered per child in a community. There are examples of countries that are beginning to transition to digital immunisation registers [73]. It might be helpful to further scale up such innovation in the African region. Furthermore, immunisation programme managers can take advantage of artificial intelligence and predictive modelling to maximise the potential of their digital immunisation register for defaulter tracking [74]. For instance, machine learning can be used to predict the likelihood of default for the second dose of measles vaccine among a cohort of children receiving vaccination in a health facility. If such information is available to immunisation-focal persons within communities, proactive measures can be instituted.

The findings from this study have several implications for policies and practices among countries in the WHO African Region. Firstly, CLD can serve as a useful tool for communicating the complexity of the implementation determinants of MCV2 which is needed by immunisation programme managers and other stakeholders for advocacy. One important area of advocacy is to mobilise broad-based investments in multicomponent systemic strategies to tackle emergent behaviours arising from the complex interaction of determinants that influence implementation. And since MCV2 is coupled with routine immunisation, the spillover effects of addressing these emergent behaviours can potentially strengthen 2YL vaccination platforms. Secondly, it highlights the importance of data on implementation context in understanding the determinants that influence the implementation effectiveness of MCV2 vaccination efforts. There is a need to rethink existing routine immunisation monitoring and evaluation frameworks through a systems thinking lens to robustly account for complexity. Indeed, layering data on implementation determinants with measles vaccination programme performance indicators across diverse communities can advance experiential learning and ensure contextual precision for programme adaption and tailoring efforts. Thirdly, the identified feedback loops expose opportunities for interventions as well as policy analysis related to measles vaccination. Nevertheless, local adaptation of the CLD through a multistakeholder consultative process is encouraged.

There are multiple limitations that should be considered when interpreting the findings of this study. There is a paucity of published literature on second dose measles vaccination in the African region as only 17 articles were included in this review. Also, these studies were from a few countries in the region. Hence, there is an urgent need for more research preferably using a mixed methods study design embedding theoretical frameworks like CFIR that is conducted in West and Central Africa including areas experiencing frequent outbreaks, affected or impacted by conflicts where the literature gaps are most apparent. Secondary data were used to develop the causal loop diagram. Many of the variables that were used to build the CLD were reported across multiple studies, and this improved the comprehensiveness of the causal statements. However, it is possible that some linkages and feedback might have been omitted. And finally, since the CLD in this study was built by the authors, there are possibilities of unconscious biases.

## 5. Conclusions

There is an urgent need for more concerted and systemic efforts to optimise MCV2 implementation in the WHO African Region. The findings from this review bring to light the complexity of the implementation determinants of second-dose measles vaccination. Understanding this complexity can guide stakeholders in policy formulation and strategy design and implementation to improve and sustain optimal MCV2 coverage across diverse settings and strengthen 2YL vaccination. The use of systems thinking can transform the implementation of MCV2 by unlocking necessary systemic innovations in multiple facets of the immunisation programme structure. The prominence of “last mile” determinants in this study calls for national immunisation programmes to pay closer attention to ensuring context-relevant and context-fit adaptations of measles vaccination efforts in the second year of life so that services can be tailored to communities to optimise demand and uptake.

## Figures and Tables

**Table 1:** Characteristics of studies included in the review.

Author	Year of Publication	Study Location	Study Design	Study Setting	Study Population
Makokha [41]	2017	Kenya	Cross-sectional, quantitative	Community-based	Caregivers of children aged 24–35 months of age
Magodi et al. [42]	2019	Tanzania	Cross-sectional, quantitative	Community-based	Caregivers of children under five years
Masresha et al. [43]	2019	Eleven countries in the WHO African Region	Cross-sectional, qualitative	Programme review	Health workers, immunisation programme managers, cold chain officers
Chirwa et al. [44]	2020	Malawi	Cross-sectional, quantitative	Community and health facility-based	Health workers and caregivers of children under five years
Muluneh et al. [45]	2022	Ethiopia	Cross-sectional, quantitative	Community-based	Caregivers of children aged less than 36 months
Munyithya et al. [46]	2022	Kenya	Cross-sectional, quantitative	Community-based	Caregivers of children under five years
Koala et al. [47]	2022	Burkina Faso	Cross-sectional, mixed methods	Facility and community-based	Caregivers of children aged 24–35 months
Chilot et al. [48]	2022	Eight countries in the African region	Cross-sectional, quantitative	Community based	Caregivers of children aged 24–35 months
Mamuti et al. [49]	2022	Kenya	Cross-sectional, quantitative	Community-based	Caregivers of children aged 24–59 months
Hailu et al. [50]	2022	Ethiopia	Cross-sectional, quantitative	Community-based	Caregivers of children less than 2 years
Tadesse et al. [51]	2022	Ethiopia	Cross-sectional, quantitative	Community-based	Caregivers of children under five years
Teshale et al. [52]	2023	Ethiopia	Cross-sectional, quantitative	Community-based	Caregivers of children aged 24–35 months
Dalaba et al. [53]	2023	Ghana	Cross-sectional, quantitative	Community-based	Caregivers of children under five years
Nchimunya et al. [54]	2023	Zambia	Cross-sectional, quantitative	Health facility-based	Caregivers of children less than 2 years
Muhoza et al. [18]	2023	Ghana	Cross-sectional, quantitative	Community-based	Caregivers of children aged 12–35 months
Demewoz et al. [55]	2023	Ethiopia	Cross-sectional, quantitative	Community-based	Caregivers of children aged 24–35 months
Ogutu et al. [56]	2024	Kenya	Cross-sectional, quantitative	Community-based	Caregivers of children under five years

**Table 2:** Level of influence of implementation determinants of second dose measles vaccination across CFIR domains in the WHO African Region.

Implementation Determinants	Level of Influence						
	Measles Vaccine	Child	Caregiver	Health Worker	Health Facility	Health System	Society
Cost of measles vaccines							
Political commitment							
Political support							
Socioeconomic status of community							
Religion							
Traditional beliefs							
Waiting time for measles vaccination in health facility							
Opening hours of health facilities							
Daily measles vaccination in health facility							
Training of health workers on second dose measle vaccination							
Availability of reference materials on second dose measles vaccination in health facilities							
Availability of recording and reporting tools for immunisation							
Measles vaccine stockout							
Cold chain capacity							
Distance to health facility							
Batching of children before opening measles vaccine vial							
Payment for home-based records							
Attitude of health workers							
Health worker knowledge of second-dose measles vaccination							
Concerns about measles vaccine wastage							
Child received routine immunisation in first year of life							
Age of mother							
Child birth order							
Knowledge of vaccine-preventable diseases among mothers and caregivers							
Knowledge of immunisation among mothers and caregivers							
Knowledge of recommended measles vaccine doses among mothers and caregivers							
Sick child							
Attitude of mothers and caregivers towards immunisation							
Mother’s employment status							
Being told to bring the child for second-dose measles vaccination							
Forgetting to bring the child for second-dose measles vaccination							
Adverse events following immunisation							
Educational status of mother							
Education status of household head							
Socioeconomic status of household							
Residing in rural area							
Attendance of antenatal care visits							
Attendance of post-natal visits							
Hospital delivery							
Possession of home-based record updated with second dose of measles vaccine							
Conduct of outreach sessions							
Public engagement on measles vaccination							
Community sensitisation on measles vaccination							
Monitoring and supervision							

Table legend: Colour codes of Consolidated Framework for Implementation Research (CFIR) domains. Outer setting domain: 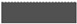; Inner setting domain: 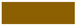; Individual domain: 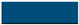; Implementation process domain: 
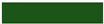
; Innovation domain: 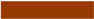.

**Table 3:** CFIR constructs of the implementation determinants of second dose measles-containing vaccination in the WHO African Region.

CFIR Domain	CFIR Construct	Identified Determinant
**Innovation**		
	Innovation cost	Cost of measles vaccines
**Outer setting**		
	Local conditions	Political commitment
	Local conditions	Political support
	Local conditions	Socioeconomic status of community
	Local attitudes	Religion
	Local attitudes	Traditional beliefs
**Inner setting**		
	Compatibility	Waiting time for measles vaccination in health facility
	Compatibility	Opening hours of health facilities
	Compatibility	Daily measles vaccination in health facility
	Access to knowledge and information	Training of health workers on second-dose measles vaccination
	Access to knowledge and information	Availability of reference materials on second-dose measles vaccination in health facilities
	Available resources	Availability of recording and reporting tools for immunisation
	Available resources	Measles vaccine stockout
	Structural characteristics	Cold chain capacity
	Structural characteristics	Distance to health facility
	Culture	Batching of children before opening measles vaccine vial
	Culture	Payment for home-based records
**Individuals**		
	Innovation deliverers	Attitude of health workers
	Innovation deliverers	Health worker knowledge of second-dose measles vaccination
	Innovation deliverers	Concerns about measles vaccine wastage
	Innovation recipient	Child received routine immunisation in first year of life
	Innovation recipient	Age of mother
	Innovation recipient	Childbirth order
	Innovation recipient	Knowledge of vaccine-preventable diseases among mothers and caregivers
	Innovation recipient	Knowledge of immunisation among mothers and caregivers
	Innovation recipient	Knowledge of recommended measles vaccine doses among mothers and caregivers
	Innovation recipient	Sick child
	Innovation recipient	Attitude of mothers and caregivers towards immunisation
	Innovation recipient	Mother’s employment status
	Innovation recipient	Being told to bring the child for second-dose measles vaccination
	Innovation recipient	Forgetting to bring the child for second-dose measles vaccination
	Innovation recipient	Adverse events following immunisation
	Innovation recipient	Educational status of mother
	Innovation recipient	Education status of household head
	Innovation recipient	Socioeconomic status of household
	Innovation recipient	Residing in rural area
	Innovation recipient	Attendance of antenatal care visits
	Innovation recipient	Attendance of post-natal visits
	Innovation recipient	Hospital delivery
	Innovation recipient	Possession of home-based record updated with second-dose of measles vaccine
**Implementation process**		
	Teaming	Conduct of outreach sessions
	Engaging	Public engagement on measles vaccination
	Engaging	Community sensitisation on measles
	Reflecting and evaluating	Monitoring and supervision
